# Prevalence of Pulmonary tuberculosis and immunological profile of HIV co-infected patients in Northwest Ethiopia

**DOI:** 10.1186/1756-0500-5-331

**Published:** 2012-06-27

**Authors:** Yitayih Wondimeneh, Dagnachew Muluye, Yeshambel Belyhun

**Affiliations:** 1 Department of Parasitology, School of Biomedical and Laboratory Sciences, College of Medicine and Health Sciences, University of Gondar, P.O. Box 196, Gondar, Ethiopia; 2 Department of Medical Microbiology, School of Biomedical and Laboratory Sciences, College of Medicine and Health Sciences, University of Gondar, P.O. Box 196, Gondar, Ethiopia; 3 Department of Immunology, School of Biomedical and Laboratory Sciences, College of Medicine and Health Sciences, University of Gondar, P.O. Box 196, Gondar, Ethiopia

## Abstract

**Background:**

In sub-Saharan Africa, as high as 2/3 of tuberculosis patients are HIV/AIDS co-infected and tuberculosis is the most common cause of death among HIV/AIDS patients worldwide. Tuberculosis and HIV co-infections are associated with special diagnostic and therapeutic challenges and constitute an immense burden on healthcare systems of heavily infected countries like Ethiopia. The aim of the study was to determine the prevalence of pulmonary tuberculosis and their immunologic profiles among HIV positive patients.

**Methods:**

A cross sectional study was conducted among adult HIV-positive patients attending HIV/AIDS clinic of Gondar University Hospital. Clinical and laboratory investigations including chest x-ray and acid fast staining were used to identify tuberculosis cases. Blood samples were collected to determine CD4+ lymphocyte count. A structured questionnaire was used to collect socio-demographic characteristics of study subjects. The data was entered and analyzed using SPSS version 16 software.

**Results:**

A total of 400 HIV positive study participants were enrolled. Thirty (7.5%, 95%CI: 5.2-10.6%) of the study participants were found to have pulmonary tuberculosis. In multivariate analysis, only CD4+ lymphocyte count (AOR = 2.9; 95% CI: 1.002-8.368) was found to be independently associated with tuberculosis-HIV co-infection. Individuals who had advanced WHO clinical stage were also statistically significant for co-infection. The mean CD4+ lymphocyte count of HIV mono-infected participants were 296 ± 192 Cells/mm3 and tuberculosis-HIV co-infected patients had mean CD4+ lymphocyte count of 199 ± 149 Cells/mm3 with p value of 0.007.

**Conclusions:**

We found high prevalence of tuberculosis-HIV co-infection. Lower CD4+ lymphocyte count was found to be the only predicting factor for co-infection. Early detection of co-infection is very necessary to prolong their ART initiation time and by then strengthening their immune status.

## Background

Tuberculosis (TB) and human immune deficiency virus (HIV) infections are two major public health problems in many parts of the world. The prevalence of TB-HIV Co-infection is higher worldwide and 90% of these co-infected cases live in developing nations [1-3]. Tuberculosis is the most common opportunistic disease and cause of the death for those infected with HIV [3]. Similarly, HIV infection is one of the most important risk factors associated with an increased risk of latent TB infection progressing to active TB disease [4,5]. In persons infected with TB only, the lifetime risk of developing TB ranges between 10% and 20% [6,7]. However in persons who have been co-infected with TB and HIV, the annual risk can exceed 10% [8-10].

An estimated 1.37 million HIV positive TB patients were diagnosed globally in 2007, and around 80% of them live in sub-Saharan Africa [11]. Tuberculosis and HIV co-infections are associated with special diagnostic and therapeutic challenges and constitute an immense burden on healthcare systems of heavily infected countries like Ethiopia [12].

Unlike other opportunistic infections which occur at CD4+ lymphocyte count below 200/mm3, active TB occurs throughout the course of HIV disease [13]. Clinical presentation of TB in HIV-infected individuals depends on the level of immunosuppression resulting from HIV infection. In patients with relatively intact immune function (CD4+ lymphocyte count >200/mm3), pulmonary tuberculosis (PTB) is more frequently seen than extra pulmonary TB [14,15].

Ethiopia is among the countries most heavily affected by the HIV and TB. The World Health Organization (WHO) has classified Ethiopia 7th among the 22 high burden countries with TB and HIV infection in the world [16].

It is recognized that joint TB-HIV interventions will clearly require additional funding to improve both TB and HIV program performance and coverage, increase testing and counseling, prevent mother to child transmission of HIV infection, provide community home based care for people living with HIV/AIDS and provide antiretroviral treatment. Despite this needs, there is no adequate and recent data in Ethiopia especially in this study area. Therefore, the aim of this study was to determine the prevalence of PTB among pre-antiretroviral treatment (ART) HIV positive patients and their immunologic profiles which is important for health professionals and policy makers to make evidence based decisions.

## Methods

### Study design, period and setting

A cross sectional study was conducted from March 27 to May 30, 2011 at Gondar University Hospital, Northwest Ethiopia. Gondar University Hospital is a teaching referral hospital located 720 km North West of Addis Ababa. It is the only referral hospital for North West Ethiopia serving a population of about 5 million coming from different geographical locations surrounding it. The hospital provides inpatient and outpatient services, including care and treatment for TB and HIV/AIDS patients with ARTs. Patients being evaluated for ART initiation undergo a routine medical examination, including screening for TB disease and other opportunistic infections.

### Source population and Study participants

The source populations for this study were all HIV positive individuals who have the access to be served in Gondar University Hospital. The study participants were all ART naïve HIV positive adult individuals who have visited ART clinic at Gondar University Hospital during the study period. A total of 422 subjects were enrolled in the study considering 95% confidence, 5% margin of error, 50% of prevalence of TB (since there was no previous estimate of TB among HIV infected individuals in the area) and 10% contingency by using single proportion formula [17]. Study subjects were selected using systematic sampling technique from patient’s record in HIV/AIDS clinic by taking patients registered in every 6^th^ interval. Those ART naïve adult HIV positive individuals who gave informed consent and enough amount of blood sample were included but Individuals who were on ART and those with known chronic illnesses like diabetes mellitus and hypertension were excluded from the study.

### Data collection procedures

All subjects underwent TB screening which include: (1) symptom screening and physical examination, (2) patients having sputum production and clinical manifestations were requested to provide three sputum specimens (spot-morning-spot) for smear microscopy, and (3) chest radiography. Study participants were interviewed about the presence of clinical manifestations of TB. Socio-demographic characteristics and other related risk factors were collected using structured questionnaire by trained nurses and physical examination was done by physicians. Posterior and anterior chest x-ray (CXR) was done for patients having sputum production and clinical manifestations and interpreted. All patients having sputum production and clinical manifestations provided 3 sputum samples for smear examinations (spot-early morning-spot). Sputum was collected in a plastic leak-proof container and smear was done for acid fast staining using a direct Ziehl-Neelson (ZN) technique [18]. Five milliliter of venous Blood was aseptically collected in ethylene diamine-tetra-acetic acid (EDTA) tubes for CD4+ lymphocyte count. The CD4+ lymphocyte count was done by BD FACS count flow cytometry machine [19]. The daily, weekly and monthly maintenance of the BD FACS count flow cytometry was done according to the instruments manual and quality control for both the reagent and the machine was done daily.

### Case definitions

Smear positive PTB was defined as one or more sputum smear examinations positive for acid fast bacilli (AFB). Smear-negative PTB was also defined as three sputum smear examinations negative for AFB but with clinical and radiographic abnormalities consistent with active tuberculosis [20].

### Data analysis

Data were checked for completeness, cleaned manually and entered and analyzed using SPSS version 16 statistical software (SPSS Inc. Chicago, USA). Data were summarized using frequency tables. Backward Stepwise logistic regression model was fitted to identify different determinants of TB-HIV co-infection. Standard techniques for model checking, including the Hosmer-Lemeshow goodness of fit test, were carried out to determine the adequacy of the regression model. Statistical significance was inferred at P-value <0 .05. Mean plus standard deviation with 95% confidence interval (CI) was also used for continuous variables and the difference in means was compared with independent-sample t-test.

### Ethical considerations

Ethical issues were approved by ethical review committee of Department of Microbiology, Immunology and Parasitology, College of Medicine and Health sciences, University of Gondar. Oral and verbal informed consent was obtained from the patients prior to enrolment. Patients having tuberculosis were directed to TB/HIV clinic for treatment.

## Results

A total of 422 study subjects were sampled but 22 of them were not included because of absence of sputum production for microscopy. Out of 400 study participants, 122(30.5%) were males (with mean age of 37 + 9 years) and 278 (69.5%) were females (mean age of 32 + 9 years) with male to female ratio of 0.4:1. The lowest and the highest age of the study participants were 18 and 70 years respectively. Three hundred thirty five (83.8%) of study participants were from urban and the rest were from rural areas. Half of the participants (50%) were married and 151 (37.8) were illiterate. Out of the total study participants, 279 (69.8%) were housewives and daily laborers [Table 1].

**Table 1:** Socio-demographic characteristics of HIV positive study participants at University of Gondar Teaching Hospital, North West Ethiopia, 2011

**Characteristics**		**Frequency**	**%**
Age	18-29	128	32.0
	30-39	167	41.8
	40-49	78	19.5
	50 and above	27	6.8
Sex	Male	122	30.5
	Female	278	69.5
Residence	Urban	335	83.8
	Rural	65	16.2
Marital status	Single	75	18.8
	Married	200	50.0
	Divorced	82	20.5
	Widowed	43	10.8
Educational status	Illiterate	151	37.8
	Elementary school	96	24.0
	High school	112	28.0
	Certificate and above	41	10.2
Occupational status	Government employed	58	14.5
	Merchants	42	10.5
	Housewife and daily laborer	279	69.8
	Students	7	1.8
	Commercial sex workers	14	3.5

### Tuberculosis-HIV co-infection

Thirty (7.5%, 95%CI: 5.2-10.6%) of the study participants were found to have PTB. Of these TB-HIV co-infected cases, 19 (63.3%) were smear negative PTB. The majority of PTB cases 27 (90%) were found to have chest radiological abnormalities consistent with active PTB and about 93.3% of co-infected study subjects were found to have constitutional symptoms; cough of >2 weeks duration, fever, night sweat and weight loss. The majority of participants 263 (65.7%) were in WHO clinical stage of I followed by WHO clinical stage II 65 (16.3%) and WHO clinical stage III 61 (15.3%). Only 11 (2.8%) study participants were found to be in WHO clinical stage IV [Table 2].

**Table 2:** Clinical and immunological profile of study participants at University of Gondar Teaching Hospital, North West Ethiopia, 2011

**Variables**		**Pulmonary tuberculosis**		**Total**
		**Yes (TB-HIV)**	**No (HIV alone)**	
WHO clinical stage (n = 72)	Stage III	21 (34.4%)	40(65.6%)	61 (84.7%)
	Stage IV	9 (81.8%)	2 (18.2%)	11 (15.3%)
Smear positive	Yes	11 (100%)	0 (0%)	11 (2.7%)
	No	19 (4.9%)	370 (95.1%)	389 (97.3%)
Chest radiography	Suggestive	27 (100%)	0 (0%)	27 (6.7%)
	Not Suggestive	3 (0.8%)	370 (99.2%)	373 (93.3%)
Constitutional symptoms (cough, fever, night sweat weight loss)	Yes	28 (22.6%)	96 (77.4%)	124 (31%)
	No	2 (0.7%)	274 (99.3%)	276 (69%)
CD4+ Lymphocyte count	<200Cells/mm3	16 (11.3%)	126 (88.7%)	142 (35.5%)
	200-349Cells/mm3	8 (5.6%)	135 (94.4%)	143 (35.8%)
	≥350 Cells/mm3	6 (5.2%)	109 (94.8%)	115 (28.7%)

### Immunological profile of study subjects

The majority of study participants 285 (71.3%) had CD4+ lymphocyte count of less than 349 Cells/mm3 and 115 (28.7%) of study participants had CD4+ lymphocyte count of greater than or equal to 350 Cells/mm3 [Table 2]. Sixteen (53.3%) of co-infected patients were found to have CD4+ lymphocyte count less than 200 Cells/mm3. The mean CD4+ lymphocyte count of HIV mono-infected participants was 296 ± 192 Cells/mm3 and TB-HIV co-infected patients had mean CD4+ lymphocyte count of 199 ± 149 Cells/mm3 with P-value of 0.007.

### Predictors of TB-HIV co-infection

In multivariate analysis, only CD4+ lymphocyte count was found to be independently associated with TB-HIV co-infection. Individuals who had CD4+ lymphocyte count of <200Cells/mm3 were 2.9 (95% CI: 1.002-8.368) times more likely to be co-infected than individuals who had CD4+ lymphocyte count of ≥350Cells/mm3. Though the independent effect (in multivariate analysis) of WHO clinical stage is not determined because of zero cells of stage I and stage II, individuals who had a WHO clinical stage of IV were 8.6 (95% CI:1.69-43.34) times more likely to be co-infected compared to individuals who had a WHO clinical stage of III in crude analysis. Government employed individuals were 56% less likely to be co-infected compared to commercial sex worker [Table 3].

**Table 3:** Predictors of TB-HIV co-infection among study participants at University of Gondar Teaching Hospital, North West Ethiopia, 2011

**Variables**	**Pulmonary tuberculosis**		**OR (95%CI)**		**P value**
	**Yes (TB-HIV)**	**No (HIV alone)**	**Crude**	**Adjusted**	
Age					
18-29	10 (7.8%)	118 (92.2%)	1		
30-39	9 (5.4%)	158 (94.6%)	0.67 (0.265-1.706)		
40-49	9 (11.5%)	69 (88.5%)	1.54 (0.596-3.973)		
50 and above	2 (7.4%)	25 (92.6%)	0.94 (0.195-4.575)		
Sex					
Male	9 (7.4%)	113 (92.6%)	1		
Female	21 (7.6%)	257 (92.4%)	1.03 (0.456-2.310)		
Residence					
Urban	28 (8.4%)	307 (91.6%)	1		
Rural	2 (3.1%)	63 (69.9%)	0.35 (0.081-1.499)		
Marital status					
Single	8 (10.7%)	67 (89.3%)	1.16 (0.329-4.119)		
Married	10 (5%)	190 (95%)	0.51 (0.153-1.720)		
Divorced	8 (9.8%)	74 (90.2%)	1.05 (0.299-3.721)		
Widowed	4 (9.3%)	39 (90.7%)	1		
Educational status					
Illiterate	10 (6.6%	141 (93.4%)	1.38 (0.291-6.575)		
Elementary school	12 (12.5%)	84 (87.5%)	2.79 (0.595-13.05)		
High school	6 (5.4%)	106 (94.6%)	1.10 (0.214-5.701)		
Certificate and above	2 (4.9%)	39 (95.1%)	1		
Occupational status					
Government employed	4 (6.9%)	54 (93.1%)	0.44 (0.073-2.713)		
Merchants	6 (14.3%)	36 (85.7%)	1.00 (0.178-5.632)		
Housewife & daily laborer	16 (5.7%)	263 (94.3%)	0.36 (0.075-1.772)		
Students	2 (28.6%)	5 (71.4%)	2.40 (0.261-22.10)		
Commercial sex workers	2 (14.3)	12 (85.7%)	1		
WHO clinical stage (n = 72)					
Stage III	21(34.4%)	40 (65.6%)	1		
Stage IV	9 (81.8%)	2 (18.2%)	8.57 (1.695-43.341)*		
CD4+ Lymphocyte count					0.042
<200Cells/mm3	16 (11.3%)	126 (88.7%)	2.31 (0.872-6.102)	2.89 (1.002-8.368)*	
200-349Cells/mm3	8 (5.6%)	135 (94.4%)	1.08 (0.363-3.196)	1.19 (0.378-3.726)	
≥350 Cells/mm3	6 (5.2%)	109 (94.8%)	1	1	

* Statistically significant (p-value < 0.05).

## Discussion

The ever-increasing prevalence of PTB in Ethiopia has been made worse by the increasing incidence of HIV/AIDS. In this study, we noted higher prevalence of PTB (7.5%) among HIV positive pre-ART patients. This finding is in line with studies conducted in Nigeria (7.8%) and Tanzania (8.5%) [21,22]. However, the finding of this study was lower compared to studies conducted in Cambodia (12%), Nigeria (32.8%) and India (19.2%) [23-25]. The presence of this difference could be explained by the fact that this study considers only PTB but not other forms of TB. Our study was also restricted to pre-ART HIV positive patients in which relatively strong immunity might have contributed for lower prevalence of TB-HIV co-infection since opportunistic infections are more prevalent during compromised immune status.

Of pulmonary tuberculosis co-infected cases, 19 (63.3%) were smear negative. This result is almost in line with study findings of Tanzania (60%), India (68.9%) and Ethiopia (58%) [22,25,26] but lower than what was reported from Nigeria (82.5%) [21]. This difference might be due to the variation in concentration of acid fast bacilli in the sputum and the rate of caseation necrosis.

Tuberculosis can occur at any stage of HIV disease, and its manifestations depend largely on the level of immunosuppression. Early during HIV disease, symptoms and signs are similar to those in HIV-uninfected persons: the lungs are most commonly affected, with cough, fever, and respiratory signs along with radiographic lesions, often with cavitations [27]. In the present study, 93.3% of co-infected study subjects were found to have constitutional symptoms; cough of greater than two weeks duration, fever, night sweat and weight loss. Radiological abnormalities suggestive of PTB are also witnessed in 90% of co-infected study subjects. This result was in parallel with previous findings used for TB diagnosis and treatment guideline development.

The mean CD4+ lymphocyte count of HIV mono-infected participants was 296 ± 192 Cells/mm3 and TB-HIV co-infected patients had mean CD4+ lymphocyte count of 199 ± 149 Cells/mm3. The mean difference was statistically significant with p value of 0.007. Sixteen (53.3%) of co-infected patients were found to have CD4+ lymphocyte count of less than 200 Cells/mm3. As CD4+ lymphocyte count decreased the body defense mechanism will be overwhelmed by various opportunistic infections. In multivariate analysis, CD4+ lymphocyte count was found to be independently associated with TB/HIV co-infection. Individuals who had CD4+ lymphocyte count of <200Cells/mm3 were 2.9 times more likely to be co-infected than individuals who had CD4+ lymphocyte count of ≥350Cells/mm3. A study conducted in Nigeria revealed similar finding where lower CD4+ lymphocyte count was observed in co-infected patients than mono infected patients [21]. Our study had also revealed a significant statistical association between WHO clinical stage and TB-HIV co-infection. Individuals who had a WHO clinical stage of IV were 8.6 times more likely to be co-infected compared to individuals who had a WHO clinical stage of III in crude analysis. Those patients with advanced WHO clinical stage had higher likelihood of having TB and other opportunistic infections as it is seen in CD4+ lymphocyte count.

Though it is not statistically significant, government employed individuals were 56% less likely to be co-infected compared to commercial sex worker. This could happen due to obvious reasons that commercial sex workers are liable for many opportunistic infections as an occupational risk. It needs great emphasis for prevention and interventional activities to reduce the burden of opportunistic infections and other complications associated with HIV/AIDS.

Our study included only pre-ART HIV positive patients which have relatively strong immunity compared to patients on ART. This needs great emphasis to avoid misapprehension by health professionals for early detection of opportunistic infections. Hence the main point of selecting only ART naïve patients in this study is to appreciate the value of close follow up of pre-ART patients to prevent early deterioration of patients by undetected opportunistic infections. Early detection of opportunistic infections including tuberculosis could be performed by lower level of health institutions including health extension workers involvement. This study was conducted at HIV/AIDS clinic and recruited those patients having regular follow up to imitate the situation present in the area. The limitation of this study is that laboratory diagnosis of TB was made only by ZN technique in addition to clinical and radiological investigations. Culture and molecular techniques were not used because of unavailability.

## Conclusion

Higher prevalence of PTB was found in pre- ART HIV positive patients. Lower CD4_+_ lymphocyte count was found to be the only predicting factor for co-infection. Early detection of co-infection is very necessary to prolong their ART initiation time and by then strengthening their immune status. Further research both on ART and pre-ART patients is recommended to know the co-infection rate.

## Abbreviations

AFB, Acid Fast Bacilli; AIDS, Acquired Immune Deficiency Syndrome; ART, Anti Retroviral Treatment; BD FACS, Becton Dickinson Fluorescence-Activated Cell Sorting; CD4, Cluster of Differentiation; CI, Confidence Interval; CXR, Chest X-Ray; EDTA, Ethylene Diamine-Tetra-acetic Acid; HIV, Human Immunodeficiency Virus; OR, Odds Ratio; PTB, Pulmonary Tuberculosis; SD, Standard Deviation; SPSS, Statistical Packages for Social Sciences; TB, Tuberculosis; WHO, World Health Organization; ZN, Ziehl-Neelson.

## Competing interests

The authors declare that they have no competing interests.

## Authors’ contributions

YW: participated in conception and design of the study, data collection, analysis and interpretations of the findings, reviewed the manuscript. DM: participated in the design of the study, analysis and interpretations of the findings, drafting the manuscript and write up. YB: supervision of the study, analysis and interpretations of the findings, reviewed the manuscript. All authors reviewed and approved the final manuscript.
